# Posterior regeneration in *Isodiametra pulchra* (Acoela, Acoelomorpha)

**DOI:** 10.1186/1742-9994-10-64

**Published:** 2013-10-28

**Authors:** Elena Perea-Atienza, Maria Botta, Willi Salvenmoser, Robert Gschwentner, Bernhard Egger, Alen Kristof, Pedro Martinez, Johannes Georg Achatz

**Affiliations:** 1 Department of Genetics, University of Barcelona, Av. Diagonal 643, edifici annex, planta 2a, 08028 Barcelona, Spain; 2 Department of Evolutionary Developmental Biology, University of Innsbruck, Technikerstrasse 25, 6020 Innsbruck, Austria; 3 Department of Genetics, Evolution and Environment, University College London, Darwin Building, Gower Street, London WC1E 6BT, UK; 4 Department of Integrative Zoology, University of Vienna, Althanstrasse 14, UZA 1, 1090 Vienna, Austria; 5 Institució Catalana de Recerca i Estudis Avançats (ICREA), Passeig Lluís Companys 23, 08010 Barcelona, Spain

**Keywords:** Stem cell, Neoblast, Copulatory organ, Epimorphosis, Morphallaxis

## Abstract

**Introduction:**

Regeneration is a widespread phenomenon in the animal kingdom, but the capacity to restore damaged or missing tissue varies greatly between different phyla and even within the same phylum. However, the distantly related Acoelomorpha and Platyhelminthes share a strikingly similar stem-cell system and regenerative capacity. Therefore, comparing the underlying mechanisms in these two phyla paves the way for an increased understanding of the evolution of this developmental process.

To date, *Isodiametra pulchra* is the most promising candidate as a model for the Acoelomorpha, as it reproduces steadily under laboratory conditions and is amenable to various techniques, including the silencing of gene expression by RNAi. In order to provide an essential framework for future studies, we report the succession of regeneration events via the use of cytochemical, histological and microscopy techniques, and specify the total number of cells in adult individuals.

**Results:**

*Isodiametra pulchra* is not capable of regenerating a new head, but completely restores all posterior structures within 10 days. Following amputation, the wound closes via the contraction of local muscle fibres and an extension of the dorsal epidermis. Subsequently, stem cells and differentiating cells invade the wound area and form a loosely delimited blastema. After two days, the posterior end is re-patterned with the male (and occasionally the female) genital primordium being apparent. Successively, these primordia differentiate into complete copulatory organs. The size of the body and also of the male and female copulatory organs, as well as the distance between the copulatory organs, progressively increase and by nine days copulation is possible. Adult individuals with an average length of 670 μm consist of approximately 8100 cells.

**Conclusion:**

*Isodiametra pulchra* regenerates through a combination of morphallactic and epimorphic processes. Existing structures are “re-modelled” and provide a framework onto which newly differentiating cells are added. Growth proceeds through the intercalary addition of structures, mirroring the embryonic and post-embryonic development of various organ systems. The suitability of *Isodiametra pulchra* for laboratory techniques, the fact that its transcriptome and genome data will soon be available, as well as its small size and low number of cells, make it a prime candidate subject for research into the cellular mechanisms that underlie regeneration in acoelomorphs.

## Introduction

Regeneration is a developmental process independent of embryogenesis through which wounds heal and lost body parts are replaced following traumatic or self-induced mutilations. It involves the reconstruction of the body at all levels of biological organisation, including correct tissue polarity, structure and form [1]. Tissues might be replaced exclusively through proliferation; a mechanism called epimorphosis. In such cases, a cluster of dedifferentiated cells or stem cells usually forms at the wound site and is referred to as a blastema. Alternatively, regeneration might occur solely through the rearrangement of existing tissue; a process called morphallaxis. However, in the majority of regeneration events, a combination of the two mechanisms, epimorphosis and morphallaxis, occurs [2].

Even though regeneration is a widespread phenomenon throughout the animal kingdom, the capacity to restore damaged or missing tissue varies greatly between different phyla and within a single phylum [2]. Interestingly, the distantly related Acoela and Platyhelminthes share a stem-cell system and regenerative capacity that are strikingly similar [3,4]. In both phyla, adult stem cells located in the parenchyma, known as neoblasts, are the only mitotically active somatic cells. Their presence and importance in development, regeneration and homeostasis is recognised in the acoel families Convolutidae [4-7] and Isodiametridae [3,4]; in the latter, it has been shown that irratiated animals, which have had all their neoblasts eliminated, die soon after the treatment [3,8].

Acoels exhibit considerable regenerative capacity after experimental amputation, with some species even able to regenerate the head and the statocyst [9,10]. A direct correlation between regenerative capacity and asexual reproduction has repeatedly been suggested [11,12]. Interestingly, all modes of asexual reproduction, including paratomy (organs form before separation), architomy (organs form after the separation of parent and offspring) and budding (offspring develop with the anteroposterior axis perpendicular to that of the parent or running parallel to it but in the opposite direction), are found in acoels (for a review see [11,13]).

Mature specimens of *Isodiametra pulchra* are about 670 μm long and partly translucent. By counting the numbers of cells in suspensions made from macerated specimens and carrying out a statistical analysis of the counts, we determined that mature specimens with an average length of 668 μm ± 77 μm consist of 8090 ± 2083 cells in total.

**Figure 1:**
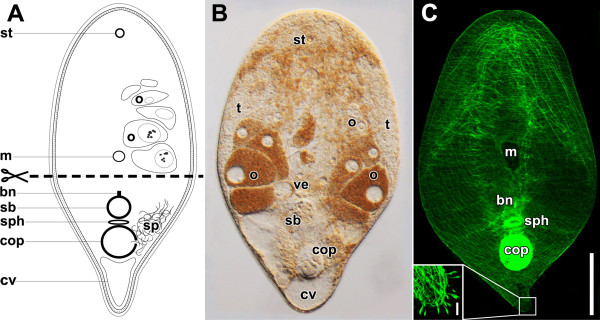
**Various representations of mature specimens of *Isodiametra pulchra*, to illustrate the internal organisation and the level of the cut.** Anterior facing upwards in all panels. **A**. Schematic drawing showing the level of the cut. **B**. Image of a live squeezed specimen. **C**. Projection of specimen labelled with fluorophore-tagged phalloidin. Inset: Posterior tip with muscle fibres and swallow’s nest receptors. Abbreviations: bn, bursal nozzle; cv, chordoid vacuole; cop, male copulatory organ; m, mouth; o, oocyte; sb, seminal bursa; sp, sperm; sph, female sphincter; st, statocyst; t, testes; ve, vestibulum. Scale bars: **A**, **B**, **C** 80 μm; **Inset** 10 μm.

The most conspicuous structures of *I. pulchra* are the statocyst at the anterior end, the prominent paired gonads extending from behind the brain towards the copulatory organs, the testes lying laterally to the ovaries, the copulatory organs and the chordoid vacuoles at the posterior end (Figure 1; [13-15]). The chordoid vacuoles are situated along the entire dorsal side of the animal but are usually only apparent at the posterior end. The mouth is situated in the centre of the ventral side and leads to a central digestive syncytium, (Figure 1A,C).

**Figure 2:**
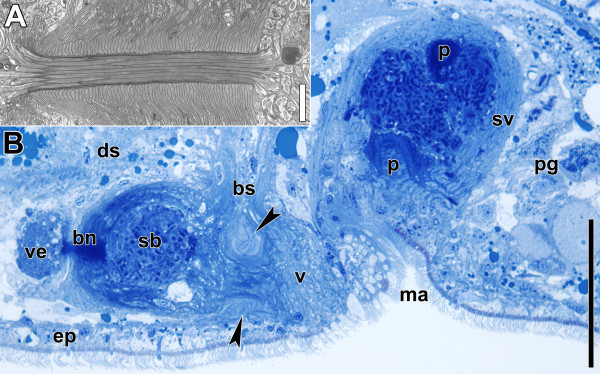
**Sagittal sections through copulatory organs of a control specimen.** Anterior is to the left in all panels. **A**. Electron micrograph of bursal nozzle. Note the sperm extending through the canal from the seminal bursa (right) into the vestibulum (left) and the numbers of cells that constitute the structure. **B**. Histological section through copulatory organs. Arrowheads point to female sphincter. Note that the connection of the vagina to the bursal stalk posterior to the sphincter is an exception and not the rule. Abbreviations: bn, bursal nozzle; bs, bursal stalk; ds, digestive syncytium; ep, epidermis; ma, male antrum; p, penis; pg, prostatoid gland cells; sb, seminal bursa; sv, seminal vesicle; v, vagina; ve, vestibulum. Scale bars: **A** 2 μm; **B** 50 μm.

The male copulatory organ is composed of a muscular seminal vesicle into which a penis is invaginated (Figures 1, 2B; [13,14,16]). The penis has the same diameter along its entire length, its lumen is lined with a specialised epithelium bearing microvilli and the musculature is made up of inner circular and outer longitudinal muscles that run strictly parallel to each other [14,16]. Prostatoid gland cells are located in the periphery of the seminal vesicle and project extensions into it (Figure 2B; [14]). Distinct gland cells open into the male antrum (the invagination around the male gonopore) and around the female gonopore (Figure 2B). Secretions from both cell types are probably functionally involved in copulation and fertilisation success.

The female copulatory organ consists of a vagina with a muscular sphincter at its proximal end. It leads into the thick-walled seminal bursa that serves to receive, store and digest sperm (Figures 1, 2; [14]). At the anterior end, the seminal bursa forms a sclerotised structure called the bursal nozzle (Figure 2; [13,14,17]). This is a thin hollow tube through which sperm have to move when passing from the seminal bursa into the vestibule to finally fertilise the oocytes [13,17,18].

At the most anterior and posterior positions on the body, swallow’s nest receptors are scattered over the surface (Figure 1C, inset; [19]). These sensory cells bear a single cilium surrounded by a collar of microvilli. The core of the microvilli and their rootlets become visible after staining with fluorophore-tagged phalloidin, as they consist of bundles of filamentous actin [19]. Unfortunately, nothing is known about the formation of the swallow’s nest receptors.

Despite the fact that it lacks the capacity to regenerate a new head [3,20], *I. pulchra* is probably the most suitable model organism to study acoel regeneration on a cellular and molecular basis. The animals are translucent, small, consiting of only about 10.000 cells (see above) and are easy to culture under laboratory conditions. Additionally, methods such as in situ hybridisation, immunostaining, 5-bromo-2’-deoxyuridine (BrdU) labelling, RNA interference (RNAi) [3,20], and high-pressure freezing (HPF) [21] have been applied successfully to them.

Some research has already used the regeneration of control and double-stranded RNA knock-down animals to infer the function of various genes, but no reference description of the regeneration process of the species was available [3,20]. Therefore, herein we determine the chronological order in which certain morphological structures (the male copulatory organ, bursal nozzle, female sphincter, and swallow’s nest receptors) appear during regeneration using light microscopy, fluorophore-tagged phalloidin combined with confocal laser-scanning microscopy (CLSM) and electron microscopy. Furthermore, we study stem cell dynamics during this process by labelling S-phase and mitotic cells, and determine the average total number of cells in adult specimens to evaluate the suitability of the species for deciphering regeneration processes at the cellular level.

## Results

### Restoration of morphological structures

#### ***Day 1***

**Figure 3:**
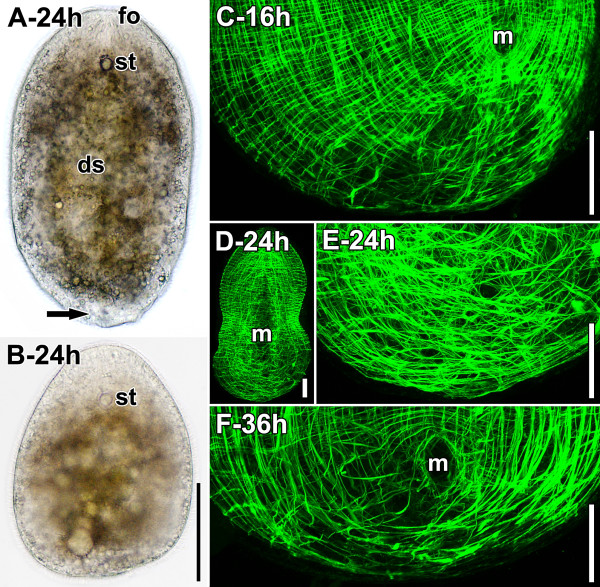
**Specimens after regenerating for 16 (C), 24 (A,B,D,E) and 36 (F) hours.** Anterior facing upwards in all panels. **A**,**B**. Live specimens. **A**. Slightly compressed specimen. Note the thickened transparent tissue on the caudal end of the animal (black arrow). **B**. Anaesthetised live specimen under coverslip. Note the blunt posterior end. **C**-**F**. Horizontal CLSM projections of specimens stained with fluorophore-tagged phalloidin (plane of optical sections perpendicular to DV axis of specimens). **C**. Posterior end of specimen. **D**. Whole specimen. **E**. Posterior end of specimen in D. Note the complete absence of any structures other than muscles and the disorganised pattern of the muscles. **F**. Ventral projection. Note that this specimen has been cut more anteriorly than the specimens in C, D and E. Abbreviations: ds, digestive syncytium; fo, frontal organ; m, mouth; st, statocyst. Scale bars: **A**, **B** 100 μm; **C**, **D**, **E**, **F** 50 μm.

Following amputation, the wound initially closes through the contraction of local circular muscle fibres and the extension of the epidermis. Consequently, the epidermis at the posterior end is thinner, the cilia are less dense and the ovaries come into contact with each other in the median plane. The closure of the wound is a very rapid process, only taking about a minute. Occasionally, a small vesicle from the digestive syncytium protrudes through the wound, but this vesicle is usually choked off within a few minutes. Consequently, a wound surface, as observed in other organisms, does not exist. After 24 hours, the wounded area shifts towards the ventral subterminal side and, in the corresponding area, a thickened translucent tissue becomes visible in live material (Figure 3A). Many cells in this half-moon-shaped area, which lies ventrally underneath the “wound epidermis”, can be determined as differentiating cells and occasionally stage 1 or 2 somatic stem cells can be found. Somatic stem cells can be distinguished by the overall organisation of the heterochromatin as well as the amount of cytoplasm and the presence of organelles [3,6,22]. The area with the highest density of differentiating cells and somatic stem cells also harbours apoptotic and necrotic cells, as well as cells of unknown identity. The tissue of the blastema is not clearly distinct from the surrounding tissue and therefore we term it a loosely delimited blastema.

Concomitant with the shift of the wound to the ventral side, the dorsal epidermis becomes thinner and the dorsoventral (DV) parenchymal muscles that usually run perpendicular to the anteroposterior body axis shift by approximately 30° towards the anterior end, adhering more frontally to the ventral body wall than to the dorsal body wall. During the first 16 hours after amputation, the original pattern of the body-wall musculature can still be recognised (Figure 3C), while after 24 hours, this pattern is no longer distinguishable (Figure 3D,E). During the first 24 hours, the posterior end maintains a roundish outline that becomes more similar to the condition of freshly cut specimens after anaesthetisation with magnesium chloride (Figure 3A,B). In specimens that are fixed in this time frame without proper anaesthetisation, the muscles contract and lose contact with the tissue they have been anchored to (epidermis, peripheral parenchyma), resulting in a posterior tip devoid of any muscle fibres. Compared to specimens that have been freshly cut, specimens that have regenerated for 24 hours are significantly smaller in terms of body width, but not length.

#### ***Day 2***

**Figure 4:**
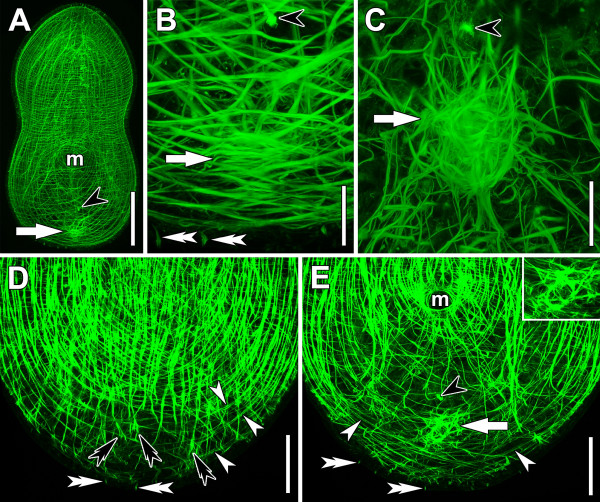
**Horizontal CLSM projections (plane of optical sections perpendicular to DV axis of specimens) of specimens after regenerating for two days stained with fluorophore-tagged phalloidin.** Anterior facing upwards in all panels. Black arrowheads point to bursal nozzle, white arrows to male primordium, white double arrowheads to swallow’s nest receptors. **A**. Whole specimen. **B**. Magnification of posterior end of specimen in A. Note the concentration of body-wall muscles and parenchymal muscles. **C**. Further developed male primordium with circular orientation on ventral side and numerous parenchymal muscles forming a distinct sphere. **D**. Dorsal projection. Note the density of circular muscles in the mid-body area and their absence at the posterior end. White arrowheads point to pairs of fibres, black double arrowheads to forked or furcated ends of muscle fibres. **E**. Ventral projection of the same specimen as in D. Note the density of circular muscles at the level of the mouth and their absence at the posterior end. White arrowheads point to pairs of fibres. Inset: Magnification showing the circular orientation of muscle fibres in the ventral part of the primordium. Abbreviation: m, mouth. Scale bars: **A** 100 μm; **B**, **C** 20 μm; **D**, **E** 50 μm.

On the second day, specimens have an egg-like shape, which they maintain even after anaesthetisation. The chordoid vacuoles that originally lay dorsally have shifted towards the subterminal tip and the angle of the DV parenchymal muscles has decreased to approximately 15° with respect to the DV axis. The body-wall musculature starts becoming more clearly structured after 36 hours post amputation (hpa). At this time, the U-shaped muscles, inner muscles, and longitudinal body-wall muscles are regularly spaced and bend around the mouth and the posterior tip on the ventral side (Figure 3F) and the dorsal side, from which the mouth and consequently the U-shaped muscles are absent. After 48 hpa, the U-shaped muscles keep this pattern whereas the other muscles are arranged in a pattern similar to that of control specimens (Figure 4A,D,E). However, the most posterior part is distinct from the rest of the body in terms of the presence and density of various muscle types (see [23,24] for terminology). Circular muscles are almost absent and cross-over muscles are sparse; but those cross-over muscles there are run continuously all the way to the lateral sides and the posterior tip (Figure 4D,E). Interestingly, they often occur in pairs, and the fibres, especially on the dorsal side, are faint. The pairs are always found to be in the same focal plane and will henceforth be termed double fibres. Most strikingly, the longitudinal muscles are completely absent and the inner parenchymal muscles are almost absent, terminating 40–80 μm anterior of the posterior tip. On the dorsal side, these muscle fibres extend slightly more posteriorly than on the ventral side, and their caudal ends are often excessively forked or furcated (Figure 4D,E). Such excessively forked or furcated terminal muscle ends have previously been interpreted as a sign of growth [25]. Strikingly, the complete re-patterning of the body-wall musculature does not apply to the area ventral of the male primordium, where the fibres maintain an unorganised orientation (see below).

The bursal nozzle is only apparent in a few specimens, but when present it is always positioned at the level where most longitudinal muscles terminate on the ventral side (Figure 4E). A differentiating female genital primordium is not apparent; however, in one specimen we observed undifferentiated cells that were arranged in a rosette surrounding an electron-dense core, which presumably would have become the bursal nozzle.

**Figure 5:**
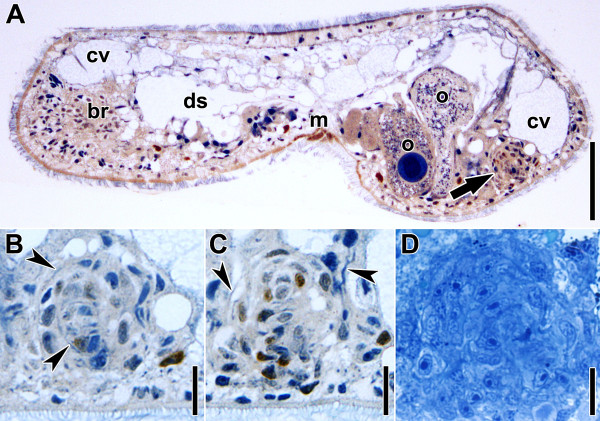
**Sagittal histological sections of specimen after regenerating for 48 hours.** Anterior is to the left in all panels. **A**-**C**. Various sections of a 1-hour-BrdU pulse specimen (cells in S-phase with orange-brownish nuclei). **A**. Whole specimen. Arrow points to male primordium. **B**,**C**. Sections through male primordium. Black arrowheads point to muscle fibres. **D**. Section through male primordium stained with Richardson’s stain. Note that the cells are arranged concentrically in a sphere but without any apparent organisation. Abbreviations: br, brain; cv, chordoid vacuole; ds, digestive syncytium; m, mouth; o, oocyte. Scale bars: **A** 50 μm; **B**, **C**, **D** 10 μm.

A spherical differentiating male genital primordium with a diameter of approximately 25 μm lies ~10 μm posterior to the nozzle when present and ~40 μm anterior to the posterior tip (Figure 5A-D). In some sectioned specimens, it appeared to be organised in an inner and an outer layer (Figure 5B,C). Thick and thin muscle fibres invading the differentiating male genital primordium and forming part of it can be observed in histological sections and through staining with fluorophore-tagged phalloidin (Figures 4A-C,E, 5B,C). The thick fibres form part of the ventral side of the primordium, whereas the thin fibres form part of its dorsal side; as evidenced in stacks from preparations using fluorophore-tagged phalloidin (Figure 4B,C,E). The thin fibres are parenchymal in position and origin, and never reach the dorsal body wall. Some body-wall muscles, inner muscles and special muscles that fan out from the posterior rim of the mouth and extend posteriorly (henceforth termed “special mouth muscles” after [23,24]) invade the primordium; however, the origin of the majority of the thick fibres cannot be determined with certainty because their orientation and position are quite unclear and many fibres in this area are forked or furcated. The distinct layering of the body wall with epidermis, body-wall musculature and peripheral parenchyma is impossible to discern in sections, because this clear pattern is extremely disorganised in the area of the differentiating male genital primordium due to the numerous differentiating cells invading the epidermis and the disorganised layering of the body-wall musculature.

As we found the musculature of the primordium in different states of organisation in various 48 hpa specimens (30% stage 0 (no obvious concentration of muscles), 30% stage 1, 30% stage 2, 10% stage 3) we determine its development as a three-step series. First, thick muscle fibres and parenchymal muscles start to concentrate in the area of the primordium, the thin parenchymal muscles fanning out in all directions except towards the dorsal side (Figure 4A,B). Secondly, the thick fibres on the ventral side form a circular pattern and the parenchymal muscles become distinct from the surrounding muscles in terms of density and thickness (Figure 4E). The muscles forming the circle lie slightly below the now distinct body-wall musculature. This arrangement of muscles correlates best with sectioned material as shown in Figure 5D, in which a compact sphere is apparent but its arrangement in two layers is not. Finally, the parenchymal muscles form a sphere, from which numerous fibres extend in all directions except towards the dorsal side, connecting it to the body wall (Figure 4C). The body-wall muscles and the muscles forming the circle are now clearly set apart and are connected through intermediate fibres. Irregularly spaced, thicker fibres start to appear in more dorsal parts of the primordium. Comparing stacks from fluorophore-tagged phalloidin preparations of this stage with histological sections, they correlate best with the material shown in Figure 5A-C, meaning that a two-layered organisation becomes apparent.

Posterior to the differentiating male genital primordium, the first swallow’s nest receptors become visible; however, the rootlets of many of them do not span the whole distance from the basal to the apical side of the cells but have started to grow from the basal side towards the apical side. The basal side of the cells can be determined because it lies close to the body-wall musculature since *I. pulchra* has an epithelial epidermis in this area (Figure 4B,D,E).

#### ***Day 3***

**Figure 6:**
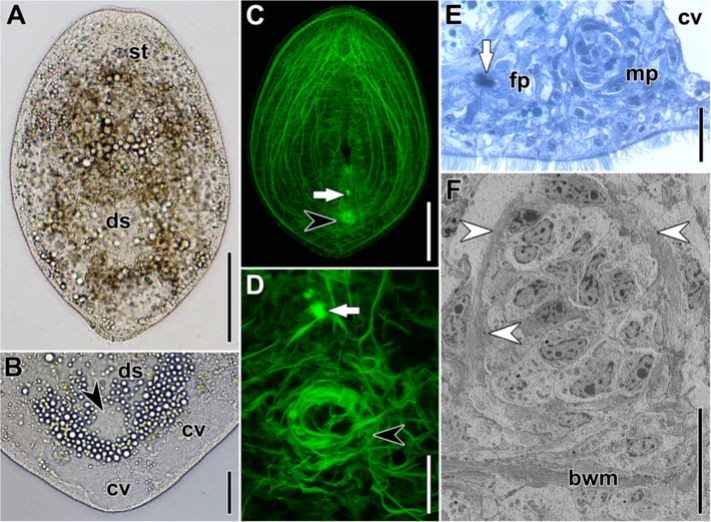
**Specimens regenerating for three days.** Anterior facing upwards in A,B,C,D and to the left in E and F. White arrows point to bursal nozzle, black arrowheads to male primordium. **A**. Slightly squeezed live specimen. **B**. Posterior end of squeezed live specimen. **C**. Horizontal CLSM projection of whole specimen. Note that the inner muscles bend away from the male primordium. **D**. Horizontal CLSM projection of primordia of copulatory organs. **E**. Sagittal histological section through primordia of copulatory organs. White arrow points to densely stained part of developing bursal nozzle. **F**. Ultrathin section of male primordium. White arrowheads point to prospective longitudinal muscle fibres of the penis. Abbreviations: bwm, body-wall musculature; cv, chordoid vacuole; ds, digestive syncytium; fp, female primordium; mp, male primordium; st, statocyst. Scale bars: **A**, **C** 100 μm; **B** 25 μm; **D**, **E** 20 μm; **F** 10μm.

On the third day, the animals have a droplet-shaped body but without a distinct tail (Figure 6A,C). The chordoid vacuoles have shifted to more posterior positions and the most posterior ones now occupy the entire space posterior to the male primordium (Figure 6B). The body-wall musculature appears completely regenerated in terms of its composition, density and extent.

Interestingly, a small bursal nozzle is apparent in all specimens studied by histological sections, but it is only apparent in a few specimens stained with fluorophore-tagged phalloidin (Figure 6C,D; Table 1). This incongruence is probably due to the fact that phalloidin only binds to filaments of polymerised actin monomers (F-actin), whereas Richardson’s stain also reveals the monomers, which are clearly present in large numbers before being assembled into polymers. The anteroposterior orientation of the nozzle is clear, but a distinct vestibulum and bursa are still missing.

**Table 1:** **Regeneration of various structures after amputation of the posterior end in *Isodiametra pulchra***


**Days**	**2**						**3**								**4**					
**Samples**	D1	D2	D3	D4	D5	**M**	E1	E2	E3	E4	E5	E6	E7	**M**	F1	F2	F3	F4	F5	**M**
**cop**	25	25	25	25	0	**20**	25	25	25	0	25	25	50	**25**	25	50	0	25	25	**25**
**sph**	0	0	0	0	0	**0**	0	0	0	0	0	0	0	**0**	0	0	0	0	0	**0**
**bn**	25	0	0	0	0	**5**	0	0	50	0	0	0	100	**21.4**	50	0	0	25	25	**20**
**# snr**	29	25	0	21	16	**18.8**	37	37	42	23	33	45	35	**36.0**	27	33	36	40	35	**34.2**
**Days**		**5**							**6**											
**Samples**		G1	G2	G3	G4	G5	G6	**M**	H1	H2	H3	H4	H5	H6	H7	H8	H9	H10	H11	**M**
**cop**		75	75	25	50	75	0	**50**	25	25	75	50	0	100	100	100	100	100	100	**70**
**sph**		0	0	0	0	0	0	**0**	0	0	0	0	0	50	50	25	0	0	0	**11**
**bn**		100	100	0	0	100	0	**50**	0	0	50	50	0	100	100	100	100	100	100	**64**
**# snr**		36	37	-	-	35	38	**36.5**	-	-	39	-	-	57	53	38	36	32	31	**40.9**
**Days**	**7**											**8**								
**Samples**	I1	I2	I3	I4	I5	I6	I7	I8	I9	I10	**M**	J1	J2	J3	J4	J5	J6	J7	J8	**M**
**cop**	100	0	100	100	100	100	100	100	100	100	**0,90**	100	100	100	100	100	100	100	100	**100**
**sph**	0	0	100	75	25	50	100	75	50	100	**58**	100	100	100	0	100	0	100	0	**63**
**bn**	100	0	100	100	50	100	100	100	100	100	**85**	100	100	100	0	100	100	100	100	**88**
**# snr**	50	20	27	40	38	-	41	-	-	-	**36.0**	-	-	39	-	50	39	46	58	**46.8**
**Days**	**9**						**10**						**controls**							
**Samples**	K1	K2	K3	K4	K5	**M**	L1	L2	L3	L4	L5	**M**	1	2	3	4	5	6	7	**M**
**cop**	100	100	100	100	100	**100**	100	100	100	100	100	**100**	100	100	100	100	100	100	100	**100**
**sph**	100	100	25	100	100	**85**	100	100	100	100	100	**100**	100	100	100	100	100	100	100	**100**
**bn**	100	100	100	100	100	**100**	100	100	100	100	100	**100**	100	100	100	100	100	100	100	**100**
**# snr**	50	56	40	44	42	**46.4**	-	-	-	42	68	**55.0**	-	53	-	56	44	48	50	**50.2**

After a given time of regeneration (bold number on top) specimens (designated as D1, D2, D3,…) were checked for the presence (100), absence (0) or grades (given as %) of differentiation of the male copulatory organ (cop), the female sphincter (sph), the bursal nozzle (bn) and the number of swallow’s nest receptors at the posterior end (# snr). Values for receptors marked with (-) signify that the receptors were too short to be counted unambiguously. The mean (M) for each day of regeneration and the control animals is given in bold in the last column for each day of regeneration. All values for day 1 (A1–A6), day 1.25 (B1–B5) and day 1.5 (C1–C5) are zero and have been omitted from the table.

The male genital primordium has grown to a size of approximately 40 μm and is slightly elongated along the proximo-distal axis. Muscle fibres are a part of the male genital primordium and are visible in fluorophore-tagged phalloidin preparations, histological sections and via electron microscopy (Figure 6D-F). Electron microscopy revealed that some of the muscles have well-organised fibres, whereas in others the filaments in the cytosol are still arranged diffusely. Nevertheless, in all the phalloidin preparations, the primordium appears to consist of a central and tube-shaped part surrounded by a dense net of parenchymal fibres. The majority of these parenchymal fibres extend laterally and some are anchored to the dorsal body wall. Many special mouth muscles invade the dense parenchymal musculature laterally but some are also situated close to it without becoming anchored. Despite also being oriented towards the primordium, the inner muscles bend away from the primordium shortly before reaching it and extend further posteriorly, attaching to the body wall (Figure 6C).

Proximally, the central part is a tube which measures 14 μm to 20 μm in length along the DV axis and consists of densely packed fibres (Figure 6D). Even though some of these fibres are oriented in a circular or longitudinal fashion, the overall appearance is irregular (Figure 6D). On the ventral side, this tube of muscles disintegrates into a disorganised network of fibres, most of which are attached to the body-wall musculature. Consequently, the ventral-most circular fibres of the muscular tube can now easily be distinguished from the ventral body-wall musculature. The measurements obtained from phalloidin preparations and electron microscopy show that the bud-like structure in Figure 6F is the primordium of the penis. With all the methods applied, the muscles forming part of the primordium of the penis appear thicker than the parenchymal fibres surrounding it (Figure 6D).

The epidermis on the ventral side of the female and male genital primordia is much thicker than in other parts of the body, and a few longitudinal body-wall muscles detach from the body wall and bend towards the male primordium. The number of swallow’s nest receptors has increased significantly (Table 1) and the rootlets of almost all of them now extend all the way from the apical to the basal side of the cells.

#### ***Day 4***

**Figure 7:**
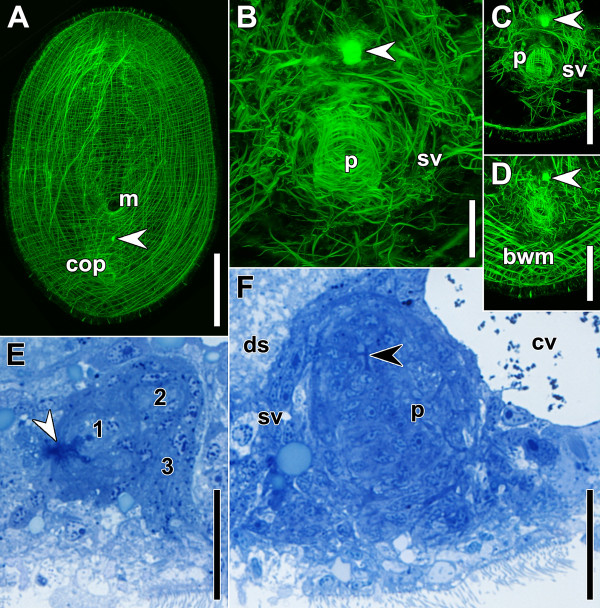
**Specimens after regenerating for four days.** Anterior facing upwards in A,B,C,D and to the left in E and F. White arrowheads point to bursal nozzle. **A**-**D**. Horizontal CLSM projections. **A**. Ventral side of whole specimen **B**. Copulatory organs. **C**. Dorsal part of specimen in B. **D**. Ventral part of specimen in B. **E**. Sagittal histological section through female copulatory organ. Note that the seminal bursa (1), the bursal stalk (2) and the vagina (3) are starting to form. **F**. Sagittal histological section through male copulatory organ. Black arrowhead points to proximal part of penis, where epithelial cells differentiate microvilli on the apical side (densely stained area). Abbreviations: bwn, body-wall musculature; cop, male copulatory organ; cv, chordoid vacuole; ds, digestive syncytium; m, mouth; p, penis; sv, seminal vesicle. Scale bars: **A** 100 μm; **B**, **E**, **F** 20 μm; **C**, **D** 50 μm.

Specimens have grown significantly and are droplet-shaped; however, a distinct tail is still missing (Figure 7A).

The bursal nozzle is now evident in the majority of specimens stained with fluorophore-tagged phalloidin (Figure 7A-D; Table 1); in histological sections, its tube-shaped structure is apparent. The vestibulum is indistinct and the seminal bursa starts to form. In general, the tissue of the female copulatory organ is distinct from the central and peripheral parenchyma surrounding it. However, in one specimen, this tissue was arranged in a “crane-shaped” pattern and the formation of the bursa, bursal stalk and vagina was apparent (Figure 7E).

The male copulatory organ has now reached a diameter of 40 μm and the length along its proximo-distal orientation of ~55 μm (Figure 7F). It is composed of a distinct penis that is thicker on the proximal than on the distal side (Figure 7B,F) and a clearly separated muscular seminal vesicle. The cells in the centre of the penis are stacked on top of each other in a circular fashion, and are more numerous in the frontal part of the penis. In a few specimens the differentiation of microvilli on the apical side of the penis epithelium could be observed in the proximal-most part of the penis (Figure 7F). The musculature of the penis consists of an inner layer of circular muscles and an outer layer of longitudinal muscles; the two types of muscles are oriented perpendicular to each other. The longitudinal muscles are arranged strictly parallel to each other and are thicker than the circular muscles (Figure 7B,C). The musculature of the seminal vesicle is distinct, but faint and loosely arranged. At the level where the muscles of the seminal vesicle and the penis converge, the longitudinal penis muscles fan out and attach to a network of fine fibres that is connected to the body-wall musculature (Figure 7D).

#### ***Day 5***

**Figure 8:**
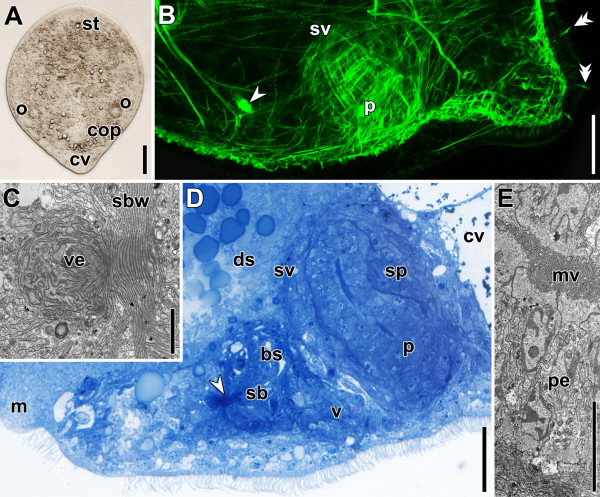
**Specimens after regenerating for five days.** Anterior facing upwards in A and to the left in B-E. White arrowheads point to bursal nozzle. **A**. Compressed live specimen. **B**. Sagittal CLSM projection through posterior end. Double arrowheads point to swallow’s nest receptors. **C**. Ultrathin section through developing vestibulum and bursal wall. **D**. Histological section through copulatory organs. Note the densely stained stripe in the centre of the penis. **E**. Ultrathin section through penis epithelium with microvilli on the apical side. Abbreviations: cv, chordoid vacuole; ds, digestive syncytium; fcop, female copulatory organ; m, mouth; mv, microvilli; p, penis; pe, penis epithelium; sbw, seminal bursa wall; sp, sperm; sv, seminal vesicle; ve, vestibulum. Scale bars: **A** 100 μm; **B** 25 μm; **C** 2 μm; **D** 20 μm; **E** 5 μm.

On day five of regeneration the animals have a typical droplet-shaped body with a distinct tail occupied entirely by chordoid vacuoles (Figure 8A,B).

The female copulatory organ has further differentiated and a subdivision into vestibulum, bursa, bursal stalk and vagina is evident in histological sections and via electron microscopy (Figure 8B-D). The vestibulum and the bursa are compact and the bursa has a distinct, multilayered wall (Figure 8C). The differentiating bursal stalk is in direct contact with the digestive syncytium, and the vagina with the body wall (Figure 8D). The male copulatory organ has further expanded and measures about 50 μm in diameter and 65 μm along its proximo-distal orientation. The muscle layer of the seminal vesicle is faint but conspicuous, even in histological sections (Figure 8B,D) and sperm can be found in the most posterior portion of it (Figure 8D). The penis has reached a diameter of ~18 μm and is bent backwards. Due to this bending, its length is difficult to determine, but it measures approximately 80 μm. All the cells in the penis epithelium have developed microvilli on the apical side and consequently an intensely stained area appears in the centre of the penis in histological sections (Figure 8D,E). The nuclei of the penis epithelium are more densely packed at the front, arranged in a zigzag pattern, being closer to the centre and the periphery in an alternating sequence (Figure 8D).

#### ***Day 7***

**Figure 9:**
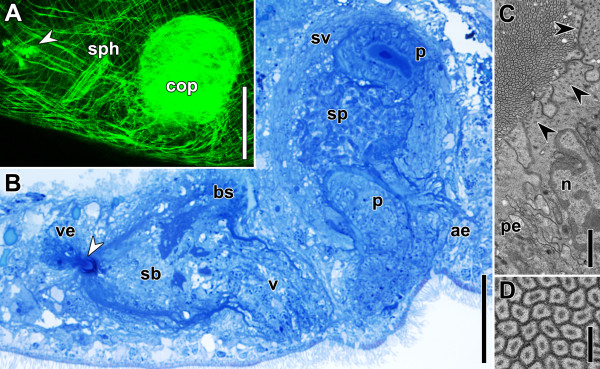
**Specimens after regenerating for seven days.** Anterior is to the left in A and B and facing upwards in C and D. White arrowheads point to bursal nozzle. **A**. Sagittal CLSM projection through copulatory organs. **B**. Semi-thin section through copulatory organs. **C**. Electron micrograph of penis epithelium and lumen. Note the numerous roots of microvilli (tiny black dots; black arrowheads) compared to Figure. 8E. **D**. Magnification of C showing cross section through microvilli with core of bundles of filamentous actin. Abbreviations: ae, antrum epithelium; bs, bursal stalk; cop, male copulatory organ; n, nucleus; p, penis; pe, penis epithelium; sb, seminal bursa; sp, sperm; sph, female sphincter; sv, seminal vesicle; v, vagina; ve, vestibulum. Scale bars: **A** 50 μm; **B** 25 μm; **C** 1 μm; **D** 200 nm.

The female copulatory organ is fully formed, with the seminal bursa and bursal stalk lined by a thick wall (Figure 9B). The vagina is in contact with the epidermis and surrounded by a weak sphincter at the proximal end. This sphincter is apparent in specimens stained with fluorophore-tagged phalloidin, but not in histological sections (Figure 9A,B). The gonopores are distinct parts of the body-wall musculature and the musculature of the male copulatory organ is fully developed (Figure 9A,B). The musculature of the antrum, penis and seminal vesicle are distinct and some of the antrum fibres are anchored along the distal part of the penis, running parallel to its longitudinal muscles. The penis is not bent but clearly curled, with a lumen starting to appear (Figure 9B,C). There are even more cells in the frontal part of the penis, but now the nuclei are positioned strictly on top of each other. The roots of the microvilli are more numerous and denser than on day 5 (compare Figure 8E with 9C). Many spermatozoa can be observed in the seminal vesicle and its muscular sheet is up to 8 μm thick (Figure 9B). Prostatoid glands are present in small numbers; they are weakly developed and do not project into the seminal vesicle. An invagination on the ventral side of the male copulatory organ, the antrum, appears in all specimens. Its epidermis is differentiated into the so-called antrum epithelium but only a small number of gland cells invade it (Figure 9B).

#### ***Day 9***

**Figure 10:**
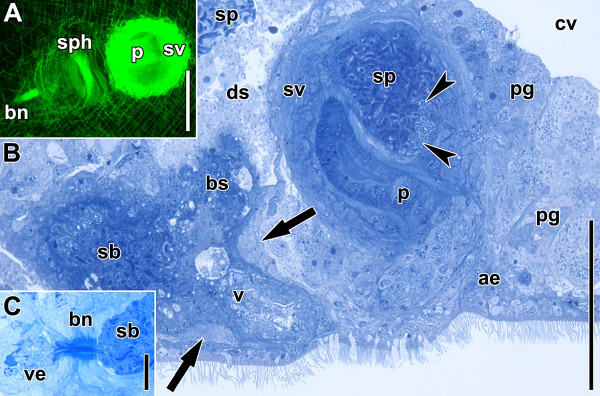
**Specimens after regenerating for nine days.** Anterior is to the lower left corner in A and to the left in B and C. **A**. Horizontal CLSM projection through central part of copulatory organs. **B**. Sagittal histological section through copulatory organs. Note the sperm in the seminal bursa and the digestive syncytium, showing that the specimen has copulated. Black arrows point to dorsal and ventral part of the female sphincter, arrowheads to distal portions of prostatoid gland cells within the seminal vesicle. **C**. Sagittal histological section through female copulatory organ. Abbreviations: ae, antrum epithelium; bn, bursal nozzle; bs, bursal stalk; cv, chordoid vacuole; ds, digestive syncytium; p, penis; pg, prostatoid gland cells; sb, seminal bursa; sp, sperm; sph, female sphincter; sv, seminal vesicle; v, vagina; ve, vestibulum. Scale bars: **A**, **B** 50 μm; **C** 20 μm.

Observations on histological sections and fluorophore-tagged phalloidin preparations suggest that the majority of specimens are now completely regenerated (compare Figure 1C and 2B with Figure 10A-C). The female copulatory organ is equipped with a strong sphincter. Numerous prostatoid gland cells surround and invade the seminal vesicle and numerous gland cells surround the female gonopore and the male antrum. However, the numbers of swallow’s nest receptors (Table 1) and gland cells suggest that regenerating animals only reach the value of the controls by day 10. Nevertheless, when kept together, specimens that have regenerated for nine days copulate, as evidenced by the sperm that is present in the seminal bursa and digestive syncytium of all the specimens studied (Figure 10B). Superfluous spermatozoa received by a partner during copulation are transferred through the bursal stalk to the digestive syncytium [13,18].

### Cell-cycle dynamics

To gain an understanding of the cellular dynamics that underlie the regeneration of morphological structures, we combined various approaches. First, we studied specimens on day one and two of regeneration by electron microscopy (see above) and performed BrdU-pulse experiments on sections to evaluate the structure of the blastema and the exact position of cells in the S-phase. Secondly, we tested whether regeneration in *I. pulchra* is one continuous process, as in most organisms, or occurs in separate waves as in some other organisms [26-28]. To this end we performed 1-hour pulse experiments on each day of regeneration and covered the entire time of regeneration with successive 12-hour continuous-labelling experiments using 5-ethynyl-2’-deoxyuridine (EdU) to make sure not to miss any peaks in proliferation.

**Figure 11:**
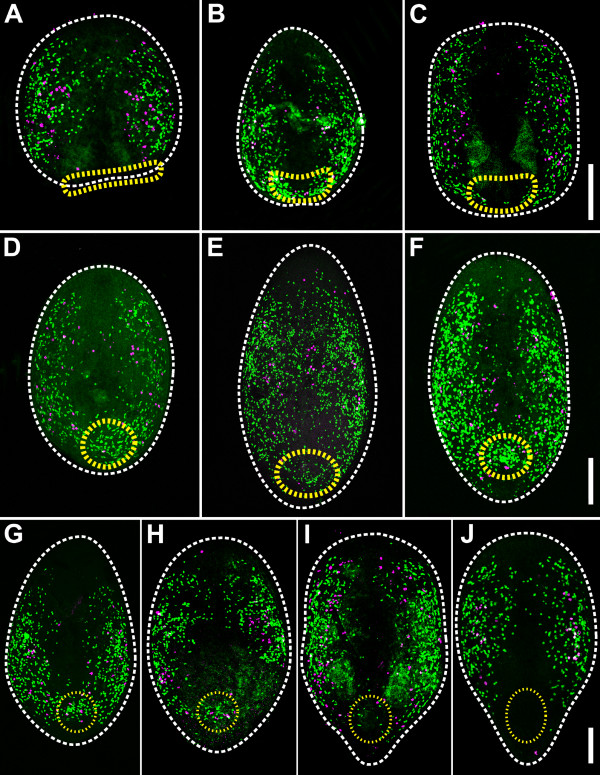
**CLSM projections showing distribution of S-phase (green) and mitotic cells (magenta) at different periods of regeneration after a 1-hour pulse with EdU.** Anterior facing upwards in all panels. White dashed lines mark the outline of the specimens. Yellow dashed lines mark: the site of the wound in A, B and C; the area of the primordia of the copulatory organs in D and E; and the area of the copulatory organs in F-J. **A**. Specimen after regenerating for 30 minutes. **B**. Specimen after regenerating for 12 hours. **C**. Specimen after regenerating for 24 hours. **D**. Specimen after regenerating for 2 days. **E**. Specimen after regenerating for 3 days. **F**. Specimen after regenerating for 4 days. **G**. Specimen after regenerating for 5 days. **H**. Specimen after regenerating for 6 days. **I**. Specimen after regenerating for 7 days. **J**. Control. Scale bars: 100 μm in all panels.

**Figure 12:**
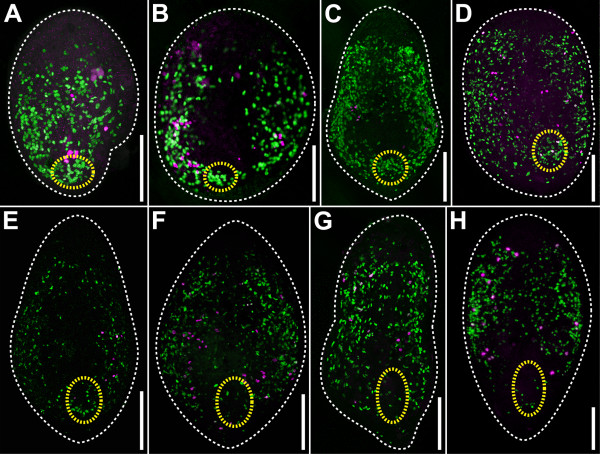
**CLSM projections showing distribution of S-phase (green) and mitotic cells (magenta) at different periods of regeneration after 12-hour continuous labelling.** Anterior facing upwards in all panels. White dashed lines mark the outline of the specimens. Yellow dashed lines mark: the site of the wound in A; the area of the primordia of the copulatory organs in B and C; and the area of the copulatory organs in D-H. **A**. Specimen after regenerating for 12 hours. **B**. Specimen after regenerating for 36 hours. **C**. Specimen after regenerating for 3.5 days. **D**. Specimen after regenerating for 4.5 days. **E**. Specimen after regenerating for 5.5 days. **F**. Specimen after regenerating for 6.5 days. **G**. Specimen after regenerating for 7.5 days. **H**. Control. Scale bars: 100 μm.

In control animals, EdU-positive cells and mitotic cells are located in two bands along the sides of the body and some isolated cells may occur in the most posterior part, however, they are less densely distributed or absent in the most anterior part and the mouth area (Figures 11J, 12H; [3]). This distribution is also found in animals after regenerating for 30 minutes, except that the posterior parts of the bands are cut off (compare Figure 11A and Figure 11J). After 12 hours, the posterior end is rounded and the two lateral bands of EdU-positive cells merge in the median plane, forming a rim below the epidermis (Figure 11B). After 24 hours, the two lateral bands become distinct again and, compared to the situation after 12 hours, only occasionally a few EdU-positive cells are found in the median area (Figure 11C). After 2 days of regeneration, the number of EdU-positive cells in this area increases; however, in line with the observations of phalloidin preparations, there is a high variability. Some specimens show no significant number of EdU-positive cells in the area of the former wound, similar to specimens after regenerating for 24 hours; whereas others harbour many EdU-positive cells (Figure 11D). Correlation with sections after BrdU incorporation (Figure 5A-C) shows that most S-phase cells in the corresponding area are part of the male genital primordium. Furthermore, these S-phase cells do not constitute a spatially defined “budding zone” but are scattered irregularly throughout the entire primordium (Figure 5A-C). Subsequently, the number of EdU-positive cells in the area of the male genital primordium or the male copulatory organ, respectively, steadily increases on day three (Figure 11E) and four (Figure 11F) of regeneration; it then starts to decrease at day five to reach the value of the controls after day 7 (Figure 11G-J).

The results of the 12-hour continuous-labelling experiments are generally in line with the 1-hour pulse experiments except that within the first 24 hpa the lateral bands of S-phase and mitotic cells are not distinct but the labelled cells are distributed almost homogeneously, only being absent from the most anterior part (compare Figure 11B,C and Figure 12A).

## Discussion

### Morphology

In general, the succession of regeneration in *I. pulchra* can be divided into three distinct but overlapping phases: an initial wound-healing phase, a crucial phase of re-patterning and a regrowth phase.

The wound-healing process is very similar to processes that take place in the acoel *Symsagittifera roscoffensis*[29], the rhabditophoran *Macrostomum lignano*[25] and freshwater triclads, which are commonly known as “planarians” [30,31]. It involves an initial contraction of circular muscles that helps reduce the surface of the wound and possibly even more importantly facilitates the establishment of new connections between epidermal cells that line the wound area [25,32]. This latter process separates the inner tissue from the surrounding medium and is indispensable for a proper course of regeneration to take place [33].

The initial reshaping of the posterior end and the shift of the wound site towards the ventral side are achieved and maintained by active muscle contraction, as evidenced by the recovery of the original “post-amputation shape” in anaesthetised specimens. In contrast to the ventral longitudinal body-wall muscles, their dorsal counterparts do not contract and are free to extend towards the caudal end. It seems highly probable that the advance in extension towards the posterior end and the occurrence of excessively forked caudal ends (that signify growth), after 48 hpa, must be caused by this difference. Interestingly, the same differences between the dorsal and ventral body-wall muscles have been found in *M. lignano* and the same reasoning has been deployed to explain them [25].

In contrast to specimens that have regenerated for just one day, which change their shape after anaesthetisation of the musculature, after regenerating for two days, specimens keep their shape, even if anaesthetised, and have a properly patterned body-wall musculature. This shows that the shape of the body is not maintained by active muscle contraction but is entirely sustained by the remodelled tissues (epidermis, muscles, parenchyma). This major transformation probably happens through the disaggregation of cell junctions and establishment of new ones. The disaggregation might explain why individual muscles of weakly anaesthetised specimens are pulled back to the mouth level without any concomitant contraction of the body wall when fixed at 16 hpa. If these muscles were properly connected to the epidermis or other tissue, such behaviour would be impossible. Additionally, the disorganised pattern of the body-wall musculature at 24 hpa (Figure 3D,E) can only be explained by a rearrangement of this musculature, and such a rearrangement requires the disaggregation and re-establishment of myoepidermal junctions (terminology after [23]) and desmosomes among muscles, or more specifically maculae adherentes and fasciae adherentes (terminology after [23]). Most strikingly, the posterior limit of the longitudinal and inner parenchyma muscles approximates the original level of the cut, thereby proving that a significant rearrangement of tissue (e.g., epidermis, chordoid tissue and peripheral parenchyma) towards the posterior tip of the regenerating worm has occurred and such a displacement and remodelling of tissue necessitates the repositioning of cells, and of course their contacts. This interpretation is further supported by the description of disorganised body-wall muscles and the disappearance of junctional complexes in the vicinity of the wound in *M. lignano*[25] and freshwater triclads [31,34].

All the processes described above are purely morphallactic events. However, some observations at the posterior end of specimens after regenerating for two days, namely the occurrence of a distinct pattern of the body-wall musculature (see below) and a thickened epidermis harbouring newly differentiated epidermal cells and swallow’s nest receptors, show that epimorphic processes also play a role in this reshaping. Interestingly, longitudinal, inner parenchymal and circular muscles are absent, while cross-over muscles extend all the way to the sides and the posterior end (Figure 4D,E). This is in stark contrast with the embryonic development of *I. pulchra*, during which cross-over muscles are the last body-wall muscles to differentiate [35]; and the sequence in *M. lignano* regeneration, where circular muscles are the first to be regenerated [25].

**Figure 13:**
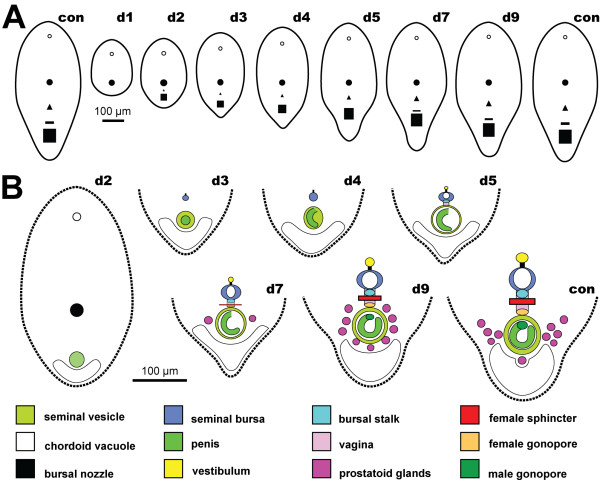
**Schematics illustrating the regeneration process in *Isodiametra pulchra *after amputation of the posterior end.** Anterior facing upwards in all drawings. Abbreviations on upper right corner of drawings denote days post amputation; con stands for control. The empty circle illustrates the statocyst; the filled black circle the mouth; the triangle the bursal nozzle; the vertical stripe positioned between the triangle and the square in controls, d5, d7 and d9 the female sphincter; and the square the male copulatory organ. **A**. General scheme of regeneration process. **B**. Regeneration process of posterior end in detail.

First, this difference might be due to the fact that cross-over muscles can be extended from the intact tissue whereas circular muscles have to be built *de novo*. Through the loss of body width on the first day of regeneration (see above, Figure 13), most probably through the shrinking of chordoid vacuoles which are distributed along the entire dorsal side of the animals, longitudinal and longitudinally orientated inner muscles do not gain any “advantage” in length. However, cross-over muscles, which span the body along this axis, do, and “superfluous” material is probably used to extend further laterally and posteriorly. Secondly, some cross-over muscles have been generated anew, as indicated by the fine appearance of some fibres and the occurrence of “double-fibres” (Figure 4D,E); a pattern reminiscent of the ontogeny of body-wall muscles, which form by using existing muscles as a template [35]. Such already existing cross-over muscles could be used as a template for newly generated cross-over muscles along the entire width of the edge of the cut; but in contrast this would only work for the most anteriorly generated circular muscle that lies adjacent to the most posterior pre-existing circular muscle.

In summary, these observations suggest that both morphallactic and epimorphic mechanisms are active in the process of early regeneration and re-patterning. The simultaneous and consecutive occurrence of both mechanisms together with a rearrangement of muscles near the site of the wound are also found in *Girardia tigrina*[34], *M. lignano*[25] and *S. roscoffensis*[29].

Despite all the commonalities in early regeneration between *I. pulchra* and *M. lignano*, it is striking that their blastemas have different appearances, specifically: loosely delimited versus compact. One difference between *I. pulchra* and *M. lignano*, besides body shape, is the presence of a digestive system consisting of wrapping cells and a digestive syncytium in *I. pulchra*[14,36] and an epithelial gut in *M. lignano*[25,37,38]. As can be seen in Figures 4, 5 and 6 in Egger et al. [38], the gut is clearly delimited at the caudal end in *M. lignano*, whereas wrapping cells and the digestive syncytium project into all regions of the body [15,37], possibly causing the loose delimitation of the blastema. Additionally, the somatic stem cells in the blastema of *I. pulchra* seem to differentiate more rapidly, also inducing a loose appearance (see below).

The accumulation of muscles in the vicinity of the blastema and the male genital primordium and their invasion is striking. A comparison of the faint and newly formed cross-over muscles with the very thick fibres in the area of the male genital primordium (Figure 4E) suggests that the thick fibres were present before the cut. Despite the uncertain identity of these thick, pre-existing muscle fibres, this interpretation is consistent with studies of *S. roscoffensis*, the genus *Convolutriloba*, *M. lignano* and *G. tigrina* in which pre-existing muscle fibres invade the newly formed blastema and serve as a template for differentiating myocytes [25,29,34,39]. Furthermore, a guiding function of these muscles has been suggested [29,34,39] and this seems to be valid in particular for the inner muscles that run from the lateral sides to the male primordium, forming a short connection between the areas that harbour most of the somatic stem cells present and the blastema or the primordia of the copulatory organs. Furthermore, the pattern of the inner muscles on days two and three and the distribution and density of parenchymal muscles indicate that the primordium of the male copulatory organ produces morphogens that attract various muscle types until day three of regeneration.

Neurite bundles (formerly termed nerve chords; terminology updated after [40]) have also been reported to invade the blastema in *S. roscoffensis* and the genus *Convolutriloba*[39,41,42] but we can neither verify nor rule out this process in *I. pulchra*, as the serotonin-like and FMRFamide-like immunoreactivity in the posterior neurite bundles of this species are too weak to monitor their regeneration and performance during the process (see [15,20] for comparison).

In line with the complete re-patterning of the body wall and internal tissues, the position of the copulatory organs is clearly determined on the second day. The male genital primordium is always distinct and the appearance of its two-layered structure indicates that some commitment, determination or differentiation of its tissue has already taken place. In contrast, the female primordium is only occasionally apparent and is solely indicated by a small bursal nozzle. However, it must be noted that the consecutive development of the female copulatory organ indicates that a female genital primordium similar to the male genital primordium must also be present; however, we probably failed to discern it because it lacks any apparent pattern or a distinct pre-existing structure that would endow it with a recognisable appearance.

Further questions might be answered through studies using the complete reconstruction of the posterior end by means of serial-section electron microscopy, the injection of various dyes to follow the movement of tissue during its rearrangement, the destruction of epimorphosis through irradiation or hydroxyurea treatment [3] and the study of apoptosis through various methods such as TUNEL assays [43]. The regeneration of the musculature might be further studied by applying antibodies that label specific subtypes of muscles, as performed on *S. roscoffensis*[29] and in experiments using expression patterns and knock-downs of instructive genes such as *IpTropomyosin*[44].

### Cell-cycle dynamics

In general, the cell-cycle experiments corroborate the morphological studies in that there is a single wave of generation and differentiation of cells. In this respect, the regeneration process is similar to that of other microturbellarians but different from the bimodal regeneration of some triclad flatworms [26,27] and an acorn worm [28]. However, the nearly ubiquitous distribution of EdU-positive cells in the first 12-hour continuous labelling and the rim of subepidermal EdU-positive cells at the posterior rim indicate that when it comes to the migration of somatic stem cells there is a first “wound-healing response” and a subsequent “re-patterning and regrowth response”.

From shortly after cutting until about 24 hpa, proliferating cells were abundant in the ventral area of the wound, where they formed a loosely delimited and half-moon-shaped blastema. Strikingly, this pattern was also shown via the mRNA expression and protein localisation of *ipiwi1*, a marker for a subpopulation of somatic stem cells [3]. However, on the third day of regeneration, *ipiwi1* mRNA expression was restricted to the genital primordia, and even though most of the BrdU-positive cells in our sectioned material occurred in the male primordium, we found a good number of BrdU-positive cells that were not directly associated with the primordium. This is another indication of the subpopulation character of *ipiwi1*-expressing somatic stem cells, but further research is needed to clarify the mechanisms of blastema and organ formation with respect to stem-cell biology.

Most remarkably, the body and its tissues were re-patterned and the genital primordia were apparent on the second day of regeneration. Importantly, BrdU- and EdU-positive cells were scattered throughout the male primordium, signifying that it was not only the body wall but also the male copulatory organ, which regenerated through intercalary growth. The presence of BrdU- and EdU-positive cells in the male genital primordium after a pulse is in stark contrast to findings for *M. lignano*, in which this organ is devoid of any such cells [38]. The same difference occurs on day three of regeneration, and furthermore, the male genital apparatus in *M. lignano* enters the phase of final differentiation, as evidenced by the formation of the stylet [38]. Consequently, individuals of *M. lignano* are already capable of copulating on the fifth day of regeneration, compared to nine days in *I. pulchra*. Taking our experiments and those of Egger et al. [38] into consideration, it seems that in *M. lignano* most of the proliferation and commitment to build up the male copulatory organ happens during the first 48 hpa, whereas in *I. pulchra* these processes take place more gradually over four to five days. Even though this difference is probably based on the large difference in the numbers of cells that make up the copulatory organ, it would be of utmost interest to determine whether acoels with a stylet (e.g. of the genera *Actinoposthia*, *Childia*, *Hofstenia* and *Paratomella*) regenerate their copulatory organ in a similar fashion to *M. lignano* and to determine constraints and differences in the regeneration of copulatory organs with hard structures between the Acoelomorpha and Macrostomorpha.

## Conclusion

Regeneration in *I. pulchra* takes place through a combination of morphallactic and epimorphic processes and the intercalary addition of structures. As in other flatworms, its succession can be divided into three distinct but overlapping phases: an initial wound-healing phase, a crucial phase of re-patterning and a regrowth phase. The closure of the wound is a very rapid process and involves the contraction of circular muscles and the extension of the epidermis. Subsequently, regenerating animals shrink in width but not length and somatic stem cells invade the wound area, forming a half-moon-shaped, loosely delimited blastema that also harbours apoptotic and necrotic cells. In contrast to the first day of regeneration, the shape of the body on the second day of regeneration does not depend on active muscle contraction but is entirely maintained by the remodelled tissue through the establishment of new cell junctions. At the same time, the body is completely re-patterned as evidenced by the appearance of the male genital primordium. EdU- and BrdU-positive cells are scattered throughout the primordium demonstrating that the male copulatory organ is also formed through intercalary regeneration. On the third day of regeneration, the body-wall musculature is complete with respect to its composition, density and extent, the female primordium is present as are complete swallow’s nest receptors. Subsequent developmental processes involve general growth and the growth and differentiation of the copulatory organs. Copulation is observed after nine days of regeneration, thereby indicating the functionality of the copulatory organs. However, the numbers of swallow’s nest receptors and gland cells suggest that animals are completely restored after 10 days of regeneration.

The regeneration sequence of the copulatory organs is as follows: 1) thick muscle fibres and parenchymal muscles start to concentrate in the area of the prospective male primordium, 2) a compact sphere appears, thick muscle fibres on its ventral side form a circular pattern and the peripheral parenchymal muscles become distinct from the surrounding muscles in terms of density and thickness, 3) the two-layered organisation of the male primordium becomes apparent and the parenchymal muscles form a sphere, from which numerous fibres extend in all directions except towards the dorsal side, and connect it to the body wall plus a female primordium with a small bursal nozzle appears, 4) a distinct separate penis (with thicker longitudinal muscles) and seminal vesicle plus a large bursal nozzle and a distinct bursa are observed, 5) the penis with distinct circular and longitudinal muscles has differentiated an epithelium that bears microvilli and is enveloped in a strongly muscular seminal vesicle plus the female copulatory organ has differentiated the vagina, bursa, bursal nozzle and vestibulum, 6) in the male copulatory organ an antrum and the gonopore are present plus in the female copulatory organ the sphincter and gonopore appear, and 7) the male copulatory organ is equipped with prostatoid glands that invade the seminal vesicle.

The suitability of *I. pulchra* for laboratory techniques, the almost complete availability of its transcriptome and genome data, as well as its small size and low number of cells, make it a prime candidate subject for research into the cellular mechanisms that underlie regeneration in acoelomorphs.

## Materials and methods

Specimens of *I. pulchra* and the diatom *Nitzschea curvillineata* (SAG, Göttingen) were kept in f/2 culture medium in a SANYO MLR-350 versatile climate chamber with the temperature set to 18°C and a light/dark regime of 14/10 hours.

For experiments, sexually mature individuals were removed from the cultures, anaesthetised with 3.5% magnesium chloride hexahydrate (MgCl_2_*6H_2_O, short form used hereafter is MgCl_2_), cut manually with a razor blade and placed individually (the anterior and posterior part of the same specimen) in different wells of 24-well cell-culture plates with filtered artificial seawater (ASW). On the following day, the posterior parts were mounted and observed with a compound microscope to determine the precise site of the cut. Only specimens with completely removed female and male copulatory organs were processed further (Figure 1A), and fed with algae twice a week with ASW changes every two days.

All the specimens processed for visualisation were anaesthetised with 7.14% MgCl_2_ before taking live images or fixation.

Live images were taken on a Leica DM 5000B compound microscope (Wetzlar, Germany) equipped with a Leica DFC 490 digital camera (Wetzlar, Germany) (Figure 1B) or a Leica MZ16F microscope (Wetzlar, Germany) equipped with a ProgRes C3 camera using a polarisation filter that was placed over the condenser and adjusted manually (Figures 3A,B, 6A).

Specimens used for preparations with fluorophore-tagged phalloidin, EdU and immunocytochemistry were fixed with freshly made 4% PFA (dissolved in 0.1 M PBS at pH 7.5). Specimens used for histology, electron microscopy and BrdU labelling were fixed following Eisenmann and Alfert, as described in [21].

Specimens for fluorophore-tagged phalloidin were processed following Achatz and Martinez ([15]; omitting steps for antibody staining) with Alexa Fluor 568 phalloidin (Molecular Probes, Eugene, OR, USA).

The number of swallow's nest receptors was determined using the software Imaris 7.2.3 (Bitplane AG, Zürich, Switzerland) by creating a 3D surface, which was subdivided into small parts of approximately the same size, segmenting only the region of interest. Subsequently, the receptors were highlighted by adjusting available parameters (e.g., area, volume, sphericity, XYZ positions). The best-fitting result was checked by eye for missing receptors, spuriously united receptors and wrongly highlighted parts, and adjusted manually; finally the receptors were counted automatically.

For cell-cycle experiments visualised with light-emitting fluorophores, the specimens were incubated for 12 h at 18°C in 125 μM EdU (Invitrogen, Camarillo, CA, USA) diluted in filtered ASW. We should mention that, based on our preliminary assessments, a concentration of 125 μM EdU is sufficient to label the S-phase cells in *I. pulchra* and that the concentration given by the manufacturer significantly increases the mortality of freshly cut specimens. Afterwards, the specimens were anaesthetised with 7.14% MgCl_2_, fixed (see above) and washed three times each with 0.1 M PBS (hereafter, PBS). Consecutively, the samples were permeabilised in a saponin-based solution, provided by the supplier, over night (o/n) and incubated in a reaction cocktail that contained 3.25 μl Alexa Flour 488 azide, 15 μl CuSO_4_, 656.5 μl EdU reaction buffer and 75 μl buffer additive for 24 h at 4°C [45] followed by three washes with PBS. Subsequently, the specimens were permeabilised and unspecific binding sites were blocked for 1 h in PBS with 0.1% Triton-X and 6% normal goat serum (Invitrogen, Camarillo, CA, USA) and incubated in a monoclonal anti-phospho-histone H3 primary antibody (Merck Millipore, Billerica, MA, USA) produced in rabbit diluted to a concentration of 1:1000 o/n at 4°C. After stringent washing in PBS with 0.1% Triton-X and blocking for 1 h in PBS with 0.1% Triton-X and 6% normal goat serum, the specimens were incubated in Alexa Fluor 568 goat anti-rabbit secondary antibody (Molecular Probes, Eugene, OR, USA) in a concentration of 1:1000 for 2 h at RT. Finally, the specimens were washed repeatedly with PBS and mounted with FluoromountG (Southern Biotech, Birmingham, AL, USA) or Vectashield (Vector Laboratories, Burlingame, CA, USA), allowing the preparations to harden o/n at 4°C.

The specimens were examined with a Leica TCS SP2 or TCS SPE confocal laser-scanning microscope (Leica Microsystems, Wetzlar, Germany).

Specimens for histological sections and electron microscopy were dehydrated in an acetone series (1 × 50%, 1 × 70%, 1 × 90%, 3 × 100%) after fixation, and embedded in EPON 812 epoxy resin (Electron Microscopy Sciences, Hatfield, PA, USA). Serial and single sections with a thickness of 0.5 μm were obtained using a diamond knife mounted in a Butler trough [46] on a Reichert-Jung Ultracut E. Semi-thin sections were stained with Richardson’s stain [47] mounted with DePeX (SERVA, Heidelberg, Germany), viewed with a Leica DM 5000B compound microscope (Wetzlar, Germany) and photographed with a Leica DFC 490 digital camera (Wetzlar, Germany). Ultra-thin sections were stained with uranyl acetate and lead citrate and examined with a Zeiss Libra 120 transmission electron microscope.

For cell-cycle experiments visualised with conventional light microscopy, the specimens were cut and left to regenerate for 24 h and 48 h. After incubating the specimens for 30 min in 5 mM 5-bromo-2’-deoxyuridine (BrdU, Sigma-Aldrich, St. Louis, MO, USA) in ASW, the medium was removed and the specimens were washed three times in ASW and anaesthetised in 7.14% MgCl_2_. Fixation was carried out in 2.5% GA in PBS and 9% sucrose at 4°C for 1 h followed by three washes in PBS and permeabilisation in PBS-T (0.1% Triton-X in PBS) for 30 minutes. Subsequently, the specimens were digested in protease XIV (Sigma-Aldrich, St. Louis, MO, USA) at RT or 37°C under inspection for about 10 min, and subsequently immersed in 0.1 M HCl for 10 min on ice, followed by 2 M HCl for 45 min at RT (48 h: 1 h at 37°C) and washed five times in PBS. Afterwards, the specimens were blocked for 15 min with BSA-T (1% bovine serum albumin and 0.1% Triton-X in PBS) and incubated with the primary mouse anti-BrdU antibody (1:600 in BSA-T, DAKO) o/n at 4°C. The following day, the specimens were washed with BSA-T, incubated for 30 min in a biotinylated goat anti-mouse/rabbit antibody, washed several times with PBS and incubated again for 30 min in streptavidin and a biotinylated horseradish peroxidase complex, with all concentrations prepared in accordance with the manufacturer’s instructions (Strept-ABComplex/HRP Duet, Mouse/Rabbit, DAKO). After washing several times in PBS and a final wash in distilled water, the specimens were developed for 1 to 2 min in a peroxidase substrate solution (DAB Chromogen, DakoCytomation), but contrary to the manufacturer’s instructions we used one drop of DAB Chromogen in 1 ml of substrate buffer and 1 ml of distilled water instead of only 1 ml of substrate buffer.

After short washes in distilled water, the specimens were dehydrated in an ethanol series (1 × 50%, 1 × 70%, 1 × 90%, 3 × 100%) and embedded in Spurr’s low viscosity resin [48] with polymerisation for 48 h at 60°C. Serial sections with a thickness of 2 μm were obtained using a Ralph glass knife mounted in a trough on a Reichert-Jung Autocut 2040 E. Sections were stained with Richardson’s stain [47] or Heidenhain’s haematoxylin [49,50], mounted with DePeX (SERVA, Heidelberg, Germany), viewed with a Leica DM 5000B compound microscope (Wetzlar, Germany) and photographed with a Leica DFC 490 digital camera (Wetzlar, Germany).

Cell numbers were determined by statistical analysis of cell suspensions. All solutions used for maceration were stored at 4°C, but used at RT. Aliquots of calcium-magnesium-free dissociation medium (modified following [6] containing 1% trypsin, 1% BSA, 0.375% Hepes, 0.027% glucose, 0.04% NaH_2_PO_4_, 0.08% NaCl, 0.12% KCl, 0.08% NaHCO_3_) were stored at -20°C and brought to RT before use. Seven to 10 specimens of similar sizes were anaesthetised with MgCl_2_, transferred to a concavity slide and measured with a reticule, which was calibrated for each magnification with a stage micrometer (C. Reichert, Austria; length 2 mm, 200 parts). After measuring, the MgCl_2_ solution was removed and 150 μl of dissociation medium were added. The specimens were then sucked up in 70 μl of the dissociation medium, transferred to an Eppendorf tube and pipetted up and down a few times. The specimens were then incubated at 37°C, checked visually every 15 min and pipetted up and down until a homogeneous cell suspension formed, which usually took 60–90 minutes. Finally, 80 μl of 0.2% DAPI (Invitrogen, Camarillo, CA, USA) in 80% glycerol in distilled water was added to the cell suspension and the suspension was mixed vigorously.

To count the cells, 20 μl of the suspension was added to the upper and lower ends (10 μl each) of a clean Bürker’s haemocytometer chamber (depth: 100 μm, area: 0.0025 mm^2^) with moistened edges of the chamber and visible Newton’s rings on the mounted coverslip. After the suspension completely filled the chamber by capillary forces, stained nuclei were counted using UV light in a Leica DM 5000B microscope. The total number of cells was calculated using the following formula: number of cells in four squares/number of specimens × 375. Cell counting for each experiment was repeated four to six times. In total, 77 specimens were used in this experiment.

The software ImageJ was used to generate projections of optical sections. Images were adjusted using levels, colour balance and curves in Photoshop CS3 and all figures were prepared using the same software except for Figure 13, which was created with Adobe Illustrator CS3. White corners that resulted from rotating the original pictures for their straight orientation in Figures 11 and 12 were obscured by the background and the outlines were drawn using Adobe Illustrator CS3.

The use of acoel flatworms in the laboratory does not raise any ethical issues and therefore approval from regional or local research ethics committees is not required.

## Competing interests

The authors declare that they have no competing interests.

## Authors’ contributions

JGA, PM, AK, RG and BE designed the project. JGA, EPA and MB cultured the animals used in the experiments. EP conducted the EdU experiments, the phalloidin stainings and the cell count with IMARIS. MB and WS conducted the cell count and the BrdU experiments including sectioning. JGA conducted the histology, electron microscopy and phalloidin stainings. JGA, EPA and PM drafted the manuscript. All the authors read and approved the final manuscript.
